# Perceived lack of behavioral control is a barrier to a healthy lifestyle in post-menopause: a qualitative study

**DOI:** 10.1186/s41043-024-00674-5

**Published:** 2024-11-05

**Authors:** Khadijeh Khademi, Mohammad Hossein Kaveh, Mahin Nazari, Abdolrahim Asadollahi

**Affiliations:** 1 Student Research Committee, Department of Health Promotion, School of Health, Shiraz University of Medical Sciences, Shiraz, Iran; 2 Research Center for Health Sciences, Institute of Health, Department of Health Promotion, School of Health, Shiraz University of Medical Sciences, Shiraz, 71536-75541 Iran; 3 Department of Health Promotion, School of Health, Shiraz University of Medical Sciences, Shiraz, Iran; 4 Department of Health Promotion and Aging, School of Health, Shiraz University of Medical Sciences, Shiraz, Iran

**Keywords:** Health, Health behavior, Lifestyle, Post menopause, Women’s health

## Abstract

**Introduction:**

Menopause is a natural phase in a woman’s life, but the quality of life and health of postmenopausal women are often compromised by unhealthy lifestyles. Therefore, it is crucial to identify the factors that influence their well-being. The main objective of this study is to explore the barriers to a healthy lifestyle among Iranian postmenopausal women.

**Methods:**

Qualitative exploratory research was conducted among postmenopausal women aged 45–65 years in three different health centers located in urban areas with varying economic level in a central city of Iran. These areas represented upscale, downtown, and downscale areas with different economic statuses (wealthy, relatively wealthy, and less wealthy). Nine focus group discussions were held, focusing on managing menopausal symptoms, physical activity, and healthy nutrition. Each topic was discussed separately in a different health center, with 10 women participating in each session. Data analysis was conducted using Graneheim and Lundman’s method.

**Results:**

The study revealed a prominent theme, “perceived lack of behavioral control as a barrier to a healthy lifestyle in post-menopause.” Two categories, “False attitudes” and “Perceived inability to engage in behavior,” were derived from 26 codes related to managing menopausal symptoms. Furthermore, a category, “Perceived inability to engage in behavior,” was formed from 11 to 13 codes related to physical activity and healthy nutrition, respectively. The theme highlighted that the perceived lack of behavioral control prevented the women from adopting a healthy lifestyle.

**Conclusion:**

Improving perceived behavioral control through the modification of attitudes and abilities is essential for maintaining a healthy postmenopausal lifestyle.

## Introduction

Menopause is a natural phase in a woman’s life that typically occurs when she is around 47–50 years old [1]. It is a phenomenon of increasing concern due to longer life expectancy. The number of postmenopausal women worldwide is expected to reach 1.2 billion by 2030 [2]. The main health concerns for postmenopausal women globally include: hot flashes lasting more than 10 years, which are the primary reason for health care providers visits [3]; cardiovascular diseases, which account for 45% of postmenopausal women’s deaths, as well as osteoporosis, cognitive decline(especially Alzheimer’s disease) [4], breast cancer as the most common cancer and the second cause of cancer-related deaths [5], sexual function disorders [3]; depression, lack of sleep, and daily fatigue [6–8]. Iranian studies have shown that up to 80% of postmenopausal women experience hot flashes, up to 86% experience night sweats, up to 94% experience vaginal dryness and avoid sexual intercourse [9], and 70% experience osteoporosis [10]. These symptoms and health issues are associated with increased healthcare costs, including hospitalizations and visits to doctors and gynecologists [11]. The total healthcare costs for these problems are estimated at $3 billion annually in the United States [12]. The more significant consequence of menopausal symptoms and health problems is a severe decline in quality of life [13, 14].

Studies have shown that the quality of life for women during menopause decreases to a moderate level, making it a significant global health concern [14–16]. Research on postmenopausal women’s quality of life in different countries has shown that 96% of them experience an unfavorable physical, mental, or social quality of life [17]. In Iranian studies, the quality of life for postmenopausal women is moderate, with menopause being described as a crisis in women’s lives [18, 19]. About 50% of women in this group are in poor health conditions due to moderate (31% of women) and severe (14% of women) menopausal symptoms [20]. Current clinical guidelines recommend menopausal hormone therapy for treating these symptoms, but it may not be suitable or desirable for all women, as it can lead to side effects [21, 22]. Therefore, it is crucial to prioritize managing these health issues by focusing on acceptability, safety, and satisfaction [23].

Research suggests that adopting a healthy lifestyle, which includes managing menopausal symptoms (e.g., home remedies), maintaining healthy nutrition, engaging in regular physical activity, and making lifestyle changes, can enhance menopausal symptoms and lead to improved health and quality of life for postmenopausal women [24–27]. However studies indicate that postmenopausal women often do not adhere to healthy behaviors, highlighting the need for more attention to promoting healthy lifestyles and understanding the factors that influence successful interventions, health care, and health policy [28–30]. While there is limited research specifically on the barriers to a healthy lifestyle during post-menopause, addressing these obstacles requires a comprehensive understanding of postmenopausal conditions and lifestyles by exploring women’s experiences [26, 29].

In summary, postmenopausal women often experience a decrease in quality of life and health problems due to their unhealthy lifestyle choices. Understanding the factors that contribute to this decline is crucial. To improve the well-being and quality of life of postmenopausal women, it is essential to recognize and overcome obstacles to healthy behaviors. Unfortunately, there is a lack of research on the challenges that postmenopausal women face when attempting to adopt healthier habits. Therefore, the main goal of this study is to identify the barriers to a healthy lifestyle among postmenopausal women in Iran.

## Methods

### Design

We conducted a qualitative study using nine single focus group discussions (FGDs) in January 2024 over a three-week period. FGDs are commonly used as a qualitative method to collect data and gain a deep understanding of social issues from a purposefully selected group of individuals rather than from a statistically representative sample of the general population. FGDs can stimulate discussion or debate on a research topic that requires collective perspectives and uncover the underlying meanings of those perspectives [31]. Therefore, to gather insightful data in our FGDs, we specifically recruited participants based on their experiences and their willingness to engage in open discussions to explore the barriers to maintaining a healthy lifestyle for menopausal women.

### Sampling strategies

**Fig. 1:**
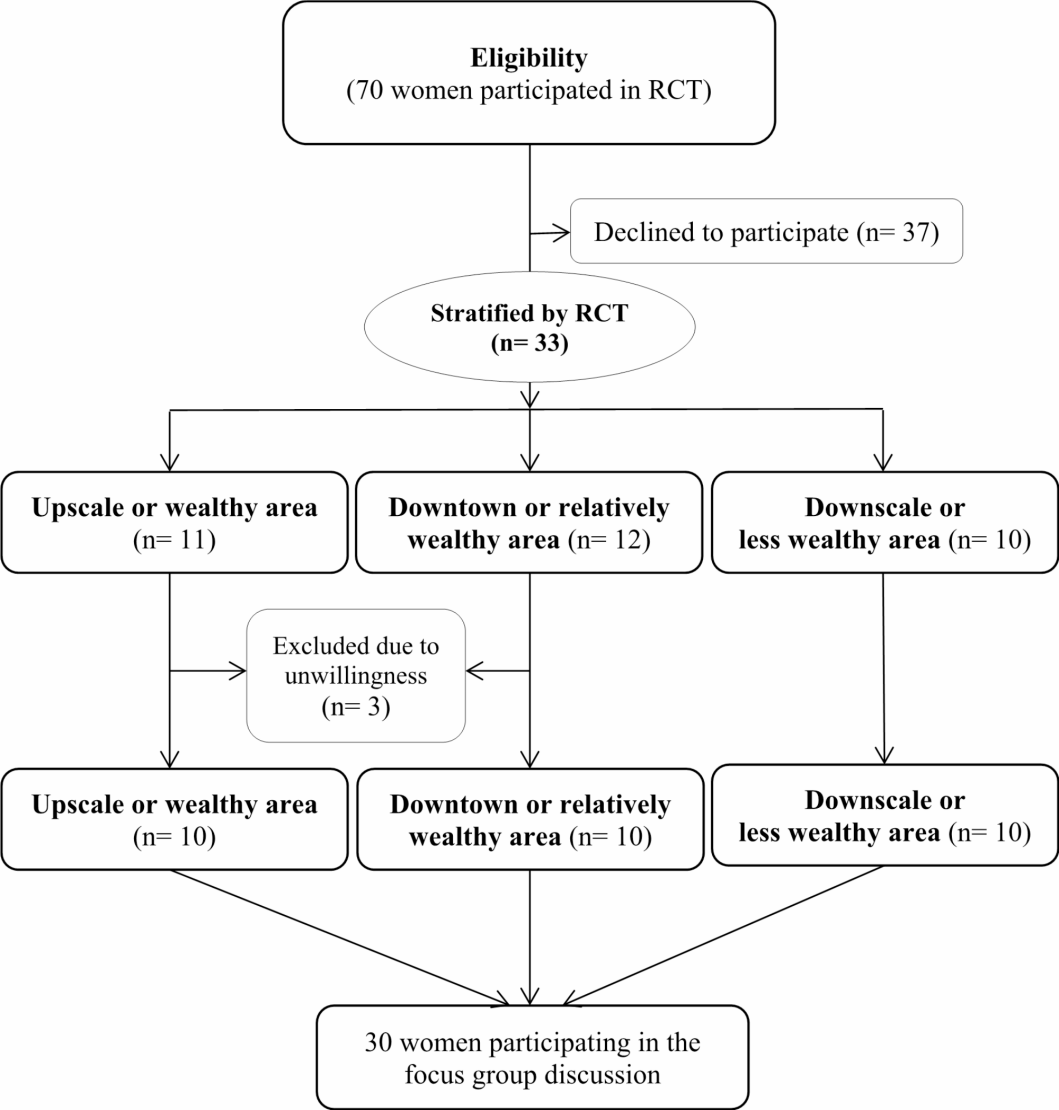
Purposeful sampling strategy for focus group discussion with postmenopausal women

A total of 30 menopausal women, divided into three groups of ten women each, were selected from three different health centers in urban areas of Shiraz. These areas were generally classified as upscale or high- income, downtown or middle- income, and downscale or low- income areas, representing different economic levels (wealthy, relatively wealthy, and less wealthy). The participants were selected using a purposeful sampling method, as depicted in Fig. 1. These women were recruited from a randomized controlled trial (RCT) study aimed at educating them about healthy lifestyles, including a healthy diet, physical activity, menopausal health, and stress control. The RCT took place from December 2023 to January 2024 and was registered under the clinical trials registration number IRCT20230716058792N1 on 14/08/2023. Seventy women actively participated in the RCT and thirty- three of them were interested in sharing their experiences.

All participants in the study were women aged between 45 and 65 years old who met the scientific definition of natural menopause, having at least one year since their last menstrual period. They also had to have a minimum literacy level of the third grade of elementary education. Participants confirmed that they were not currently undergoing psychiatric treatment for mental illnesses, did not have chronic diseases such as diabetes, hypertension, heart, or kidney failure, or cancer, and had not received hormone replacement therapy for menopausal symptoms in the past 6 months. Furthermore, they had no history of hysterectomy or oophorectomy surgery and did not experience abnormal vaginal bleeding. Please refer to Table 1 for participant characteristics. Unwillingness to continue participation was considered an exclusion criterion.

**Table 1:** Demographic data of participating women (*n* = 30)

Characteristics of participants	Frequency (%)
Age	
< 55 ≥ 55	56.7 43.3
Age at menopause	
< 50 ≥ 50	63.3 36.7
Marital status	
Married Widow	90 10
Education level	
< Diploma ≥ Diploma	46.7 53.3
Employment status	
Housewife Employed Retirement	73.3 6.7 20
Family Income sufficiency	
Less than expenses Equal to expenses More than expenses	53.3 36.7 10

### Data collection

Three FGDs were conducted to explore participants’ experiences regarding barriers to a healthy lifestyle. The discussions focused on menopausal symptom management, healthy diet, and physical activity, with each topic discussed in a separate session within each group. The discussions took place in an education room at the health centers and lasted approximately two hours. Two female researchers actively participated in the discussions, with one serving as the moderator. The moderator, the first researcher, is a PhD candidate in health education and promotion with a master’s degree in midwifery and experience in conducting interviews. She facilitated the discussions. An observer, the third researcher, is an associate professor and the lead author of several qualitative studies with a PhD in health education and promotion. She assisted in the discussions. The discussions were recorded with consent using a mini voice recorder, and the observer took brief notes on each participant’s comments in response to semi-structured, open‐ended questions. The FGDs began with introductory questions related to menopaual symptom management, such as:

- *“When were you diagnosed with menopause?“*.
- *“Which menopausal symptoms do you experience?“*.
- *“How do menopausal symptoms impact your daily life?“*.
- *“What steps have you taken to manage the symptoms of menopause?”*

For the physical activity topic, the questions were:

- *“What is the correct definition of physical activity?“*.
- *“How many days a week do you engage in physical activity?“*.
- *" How long do you engage in physical activity each day?*

Regarding healthy nutrition, the questions included:

- *“What are the food groups?“*.
- *“What is the correct definition of a daily healthy diet?“*.
- *“Do you follow a healthy diet?”*

The closing question, “Is there any other information you want to share with me?” was answered at the end of each session.

### Methods of analysis

The discussion was transcribed verbatim, and a content analysis was performed on the Persian transcripts before translation. Following Graneheim and Lundman’s method, the analysis process consisted of the following steps: (1) The recorded interviews were transcribed and read to gain an overall understanding. (2) The text was divided into meaningful units. (3) The meaningful units were extracted and encoded. (4) The initial codes were classified into subcategories based on their similarities and differences. (5) The subcategories were sorted and abstracted into categories. (6) Themes were created to link the underlying meanings in the categories [32].

During the open coding stage, all transcripts were carefully read multiple times to note participants’ experiences regarding barriers to a healthy lifestyle for menopausal women. Fifty basic codes were obtained, including 26 codes related to managing menopausal symptoms, 11 codes related to physical activity, and 13 codes related to healthy nutrition. These codes were then compared with all extracted data to identify similarities and differences. Subsequently, categories, subcategories, and themes were created (refer to Table 2).

**Table 2:** Examples of the analysis process in the topic of management of menopause

Meaning unit (statements corresponding to the aim of the study)	Condensed meaning unit	Code	Sub-category	Category
I cannot control my hot flashes at parties because when I’m invited, I have to wear party clothes made of synthetic fabrics. Cotton clothes wrinkle quickly and don’t have exciting designs; they make me look ugly.	I can’t control my hot flashes at parties because it is important to me to look beautiful and handsome.	Caring more about others’ opinions than understanding your own state	Ignoring oneself	False attitudes
I sleep in a cool place and use a light blanket for three nights, but I still experience night sweats. I wake up frequently and struggle to fall back asleep. In my opinion, the only way to reduce menopause symptoms is through medical treatment.	I avoided heat for three days in an attempt to decrease my night sweats, but it wasn’t effective. In my opinion, only medication can truly reduce them.	Applying menopause management strategies in a short amount of time and seeing no results	Negative attitudes towards actions	
Pouring cool water on my body helps reduce my hot flashes. However, when I am outside of the house and experience hot flashes, I am unable to take a shower or wash my hands and face. This can be bothersome when my hot flashes occur.	When I’m out of the house and experience a hot flash, I don’t have any way to alleviate it.	Lack of access to facilities	Inability to perform in challenges	Perceived inability to engage in behavior
I have a habit of taking a short nap after lunch to help me fell less tired and be more alert for the rest of the day, even though I tend to stay up late at night.	I have a habit of napping during the day to recharge, but I struggle with insomnia at night.	Getting used to solving problems in the wrong ways	Persistence of unhealthy habits	
As a religious person, I find it difficult to maintain tranquility when I experience hot flashes during prayer. It is a religious rule that I must not remove my hijab or abruptly stop praying.	When I pray and experience hot flashes, I struggle to find a solution to achieve tranquility.	Not making decisions calmly	Disinclination	

### Methods of quality assurance

Member checking, peer debriefing, investigator triangulation, and cross-examination were used to ensure the trustworthiness, dependability, and credibility of the data. During member checking, each participant received a transcript of their coded words and was asked if the codes accurately reflected their experiences to validate the interpreted findings (credibility). Peer debriefing was conducted by the second and fourth researchers, who held frequent sessions with supervisors to report on and discuss the study’s progress and process to ensure accuracy (credibility). Additionally, some health education and promotion faculty members, who were the main authors of several qualitative studies, checked the encoding process and categories access (investigator triangulation for trustworthiness). Lastly, cross‐examination involved comparing participant’s responses or descriptions to similar situations or conditions to establish dependability.

### Ethics

The study protocol was approved by the Ethics Committee of Shiraz University of Medical Sciences (IR.SUMS.REC.1402.049). Before the study commenced, participants were informed about the aim and procedure of the study and their right to withdraw their participation at any time. The researchers assured them of their anonymity, and written informed consent was obtained from each participant. Participation was strictly voluntary. This study complies with the consolidated criteria for reporting qualitative studies (COREQ) checklists [33].

## Result

The overall theme that emerged from the study was that the “perceived lack of behavioral control is a barrier to a healthy lifestyle in post-menopause” among Iranian women. This theme is associated with two qualitatively different categories and five subcategories related to menopause management, as well as one qualitative category and two subcategories related to physical activity and healthy nutrition (refer to Table 3). The theme is based on women’s perceived inability to engage in behaviors. This perceived lack of behavioral control process leads to the persistence of unhealthy habits and the inability to face challenges. The categories are described, along with quotes, to illustrate the range of experiences and ideas articulated by different participants. Participants’ careers are indicated within brackets.

**Table 3:** Themes, categories, and sub-categories

Perceived lack of behavioral control is a barrier to a healthy lifestyle in post-menopause		
Topic	Category	Sub-category
Management of menopausal symptoms	False attitudes	Ignoring oneself
		Negative attitudes towards actions
	Perceived inability to engage in behavior	Inability to perform in challenges
		Persistence of unhealthy habits
		Disinclination
Physical activity	Perceived inability to engage in behavior	Inability to perform in challenges
		Disinclination
Healthy nutrition	Perceived inability to engage in behavior	Inability to perform in challenges
		Persistence of unhealthy habits

### False attitudes

The category of “False attitudes” refers to beliefs that hinder the proper management of menopausal symptoms. This includes beliefs that certain strategies are useless or ineffective.

### Ignoring oneself

Women stated that they hadn’t followed certain menopause management strategies in order to appear attractive and energetic to others.

*I always wear jeans for my daily commute in an effort to look handsome*,* even though I know that this type of pants is not suitable for my hot flashes. (Retirement*,* upscale area)*

*I drink two shots of coffee daily in the morning and afternoon because I want to feel energetic at home and in the gym and not feel inferior to younger women. (Housewife*,* upscale area*,* suffering from insomnia)*

Furthermore, women mentioned that they had given up certain menopause symptom management strategies in order to gain approval for their behavior and speech from others.

*My sons buy me fast food and carbonated drinks for dinner*,* so I don’t upset them. I thanked them*,* and we ate dinner together. (Housewife*,* downscale area*,* suffering from severe hot flash)*

*I am very religious and I suffer from severe hot flashes. However*,* in our neighborhood*,* I cannot take off my hijab and wear lighter clothes because I am well- known. (Housewife*,* downscale area)*

Finally, for most women, the priority in their lives was others, as taking care of their responsibilities was more important than following menopause management strategies.

*My priority in life is my two children*,* so I am constantly cooking*,* doing laundry*,* tidying the house*,* and taking care of other household chores. I don’t have time to use any strategies and always feel tired and moody. (Retirement*,* upscale area)*

### Negative attitudes towards actions

Women reported that the strategies were not effective in reducing their menopausal symptoms. It should be noted that most women who were dissatisfied either did not follow the strategies correctly, only tried them for a short period, or only used a limited number of strategies to alleviate their symptoms.

*I suffer from hot flashes. I wear cotton clothes*,* but my symptoms persist. I don’t believe there is any way to improve hot flashes. (Housewife*,* downscale area)*

*I experience urinary incontinence when laughing or coughing*,* and I tried doing Kegel exercises- 5 contractions twice a day for 2 weeks. However*,* the incontinence did not improve*,* and there was no change. (Housewife*,* downscale area)*

*I tried to avoid napping during the day for a week to combat my insomnia but it persisted at night. I felt tired during the day*,* so I started taking a nap for an hour during the day again. (Retirement*,* downtown area)*

### Perceived inability to engage in behavior

The category of “Perceived inability to engage in behavior” consists of the inability to maintain a healthy lifestyle, which includes healthy nutrition, physical activity, and managing menopausal symptoms. This inability stemmed from challenges in certain situations, difficulty in breaking bad habits, and a reluctance to adopt healthy behaviors.

### Inability to perform in challenges

Many women expressed that it was difficult, and sometimes even impossible for them to engage in physical activity, maintain a healthy diet, and manage menopausal symptoms when they were away from home. This included attending parties, going on trips, having picnics or shopping.

*When I travel*,* I consume fast food and carbonated drinks daily because I don’t cook. (Housewife*,* upscale area)*

*When I’m out*,* such as shopping or going to the doctor’s office*,* I struggle to drink enough water*,* which leads to a burning sensation my urinary area. (Retirement*,* downtown area)*

Additionally, according to women’s statements, having guests and a lot of housework could hinder healthy behaviors.

*When I have guests*,* I can’t go to the bathroom regularly*,* and it becomes difficult for me to control my urinary incontinence. (Housewife*,* downscale area)*

*I have so many chores at home that I’m so tired I can’t sleep easily. (Housewife*,* downscale area)*

*When I have guests*,* I’m busy with catering and cleaning the house since morning*,* so I can’t do any physical activity. (Housewife*,* downtown area)*

Finally, based on reports from women experiencing physical problems such as musculoskeletal pain, stomach aches, and headaches, as well as mental concerns like worry, anger towards others, separation from loved ones, and financial worries, maintaining a healthy diet, engaging in physical activity, and following menopause management strategies became challenging or even impossible.

*I am allergic to lubricant during sex*,* so I choose not to use it. However*,* I experience pain during sex and my vagina burns for a day. (Housewife*,* downscale area)*

*Whenever I eat vegetables and dairy products*,* I experience stomach pains and feel nauseous*,* so I avoid consuming these foods. (School service driver*,* downscale area)*

*When I have back or neck pain*,* I refrain from going for walks or exercising. (Housewife*,* downtown area)*

*My husband doesn’t show me much support or love. I often dwell on this issue at night*,* which makes it hard for me to sleep and causes me to feel moody throughout the day. (Housewife*,* upscale area)*

### Persistence of unhealthy habits

Despite being aware of the negative impact that poor nutrition and ineffective management of menopausal symptoms can have on their health, women continued to engage in these habits. This was due to the difficulty of breaking away from these habits or finding it challenging to adopt healthier alternative behaviors.

*I have a strong inclination towards consuming sweets*,* which makes it hard for me to give them up. (Retirement*,* upscale area)*

*I often find myself using my phone late at night to keep my mind occupied and tire myself out*,* which in turn helps me fall asleep more easily. (Housewife*,* downscale area)*

### Disinclination

Feeling bored, lazy, overwhelmed, distracted, and restless were commonly cited as barriers to engaging in physical activity and adhering to menopause management strategies.

*When I go out to have fun with my friends*,* I get restless and excited*,* so I have trouble sleeping at night. (Housewife*,* upscale area)*

*I find it hard to get in the mood for physical activity because it takes me half an hour just to get ready to leave the house. (Housewife*,* downscale area)*

## Discussion

The findings of the present study on postmenopausal women in Iran indicate that a perceived lack of behavioral control acts as a barrier to adopting healthy behaviors and lifestyles. This finding aligns with the result of meta-analysis conducted by Cheng et al. which encompassed various cultures and societies. Their study showed that individuals with a strong health control orientation were more likely to engage in health-enhancing behaviors such as exercise (*r* = 0.10) and diet (*r* = 0.08) [34]. Similarly, Botha et al., drawing from the Household, Income, and Labour Dynamics in Australia (HILDA) survey, found that internal health control significantly impacts individual’s physical and psychological well-being, ultimately influencing their overall health outcomes [35]. To enhance postmenopausal women’s health control, it is crucial to empower them through interventions focused on healthy lifestyle choices and menopausal health. This empowerment can enable women to better manage their symptoms and make informed decisions about their healthcare [23].

In the present study, women did not adhere to certain menopause management strategies because they prioritized the opinions, attitudes, and care of others, and believed that these strategies would not alleviate their menopausal symptoms. This behavior falls under the category of “*False attitudes.”* Similarly, Vincent et al., in their systematic review, found that a higher frequency, intensity, or number of menopausal symptoms were significantly associated with greater body image concerns (e.g., self-perceived appearance, attractiveness, and body functioning) in postmenopausal women [36]. Additionally, in line with the subcategory *“Ignoring oneself*,*”* Ilankoon et al. demonstrated that taking care of family needs was crucial for the postmenopausal women in their study as they often focused on others. They concluded that in collectivistic cultures with strong family ties, where women traditionally have primary responsibilities for home and childcare, there is a risk of ignoring menopausal symptoms [2]. This behavior, categorized as *“Ignoring oneself*,*”* is a “*False attitude*,*”* that can worsen menopausal symptoms. This information can be valuable for future research and help family members and healthcare workers provide better support to women during this significant life stage [36].

Moreover, women also believed that the strategies for managing menopausal symptoms were not beneficial, as shown in the subcategory *“Negative attitudes towards actions.”* This was considered as “*False attitudes”* because they used the strategies in the short term, in incorrect or limited use conditions. Additionally, in the study by Berga et al., attitude was considered an essential factor in behavioral performance [37]. Beliefs about the outcomes of behaviors and the value of these outcomes affect the intention to perform behaviors and guide them [38]. Furthermore, many RCT and meta-analysis studies have shown that these strategies can reduce the severity of menopausal symptoms like hot flashes, sleeping disorders, urinary incontinence, etc [3, 39–41]. To increase awareness about menopause and symptom management, women should be informed. Also, educational strategies and public health practices may be beneficial for women in managing menopausal symptoms and changing lifestyle habits [1, 37].

Women faced challenges in performing symptom-relieving actions and healthy behaviors due to the convenience of unhealthy habits, and their unwillingness to change. This was evident in the category of “*Perceived inability to engage in behavior*,*”* and its subcategories “*Inability to perform in the challenge*,*” “Persistence of unhealthy habits*,*” and “Disinclination.”* Similarly, Kafaei-Atrian et al. demonstrated in their RCT that empowering and enhancing self-efficacy could improve menopausal women’s self-care. This improvement can assist health professionals in educating postmenopausal women about self-care in menopausal complications [42]. Additionally, the RCT study by Noori et al. showed that empowerment programs for women led into significant improvements in health-promoting behaviors. This suggests that similar programs can ultimately enhance women’s health [43]. Empowering postmenopausal women to have a significant perceived behavioral control can reduce barriers to healthy behaviors and self-care [23]. Therefore, a systematic review and meta-analysis of 104 studies in Africa, Asia, and America recommended that program designers and implementers should target specific empowerment outcomes, promote social capital and exchange, and tailor intervention components to desired empowerment-related outcomes [44]. Moreover, socioeconomic status plays a significant role in healthy behaviors and self-care, as these factors impact women’s ability to engage in health activities, afford medical care and housing, and manage stress. They also interact with or confound relationships between other variables and health [17–20].

The findings of this study reveal various perspectives on the healthy lifestyle behaviors of postmenopausal women and why they struggle to manage menopausal symptoms. Additionally, it sheds light on the challenges faced by postmenopausal women in Iran. This information is crucial because educational interventions addressing menopausal issues and potential symptoms relief methods must be tailored to suit the prevailing sociocultural environment. This environment includes attitudes towards aging and the demands of a busy lifestyle intertwined with family responsibilities. One of the strengths of this study is the inclusion of women from three different socioeconomic regions who had undergone a RCT. However, the transferability of the findings may be limited for several reasons. The study was conducted in a single city in Iran, and the majority of participants were housewives who were married.

## Conclusions

Perceived lack of behavioral control was identified as a barrier to postmenopausal women adopting healthy behaviors or maintaining a healthy lifestyle. This may be attributed to false attitudes and a perceived inability to engage in healthy behaviors. The study’s findings indicate two key areas in which women struggle with self- care: managing menopausal symptoms and adhering to a healthy lifestyle. The first issue is a lack of knowledge, while the second is a lack of empowerment. To address these challenges, the researchers recommended that healthcare professionals focus on empowering women through personalized care, education, and interventions. These strategies can significantly improve the quality of life for postmenopausal women. Additionally, future research should combine qualitative insights into the barriers faced by this population when adopting healthy lifestyles with quantitative data.

## Data Availability

The datasets used and/or analyzed during the current study are available from the corresponding author upon reasonable request.

## Abbreviations

HILDA: Household, Income and Labour Dynamics in Australia
RCT: Randomized Controlled Trial
